# Placing anurans in water can improve photo-based individual identification

**DOI:** 10.1371/journal.pone.0341460

**Published:** 2026-01-30

**Authors:** Edina Nemesházi, Zsanett Mikó, Nikolett Ujhegyi, Andrea Kásler, Nadine Lehofer, Veronika Bókony

**Affiliations:** 1 Konrad Lorenz Institute of Ethology, Department of Interdisciplinary Life Sciences, University of Veterinary Medicine, Savoyenstrasse 1a, Vienna, Austria; 2 Department of Evolutionary Ecology, Plant Protection Institute, HUN-REN Centre for Agricultural Research, Nagykovácsi út 26-30, Budapest, Hungary; 3 Department of Systematic Zoology and Ecology, ELTE Eötvös Loránd University, Pázmány Péter Sétány 1/C, Budapest, Hungary; 4 Institute for Wildlife Management and Nature Conservation, Department of Wildlife Biology and Management, Hungarian University of Agriculture and Life Sciences, Páter Károly utca 1, Gödöllő, Hungary; 5 Doctoral School of Biology, Institute of Biology, ELTE Eötvös Loránd University, Pázmány Péter Sétány 1/C, Budapest, Hungary; 6 Department of Zoology, Faculty of Natural Sciences, Comenius University in Bratislava, Ilkovičova 6, Bratislava, Slovakia; Tshwane University of Technology, SOUTH AFRICA

## Abstract

Identification of individuals across time and space is required for investigating numerous evolutionary-ecology and conservation-related questions, and photo-based identification is commonly used for a broad taxonomic range. Success of available photo-matching software may greatly depend on image quality as well as the focal body parts. We tested the hypotheses that individual identification by colour patterns can be facilitated by taking into account the natural medium surrounding the animals and the natural body posture they tend to take. We optimised photography methods enabling individual identification by whole-body assessment of agile frogs *(Rana dalmatina)*, and compared the reliability of those photography methods for computer-assisted identification in the HotSpotter software as well as for observers operating it. We found that photographing either hand-restrained frogs with towel-dried skin, or frogs moving freely in clean water enabled comparison of the dorsal surface of the whole body including the hind legs, and HotSpotter identified matching images at rates similar to those anuran studies that reported high success before. Specifically, the true match ranked in the top 10 for >92% of photographs taken with the above methods. By contrast, submerging hand-restrained frogs in water significantly improved identification: images of the same individual were always ranked as the most likely match. We attribute this outstanding performance to the combination of advantageous effects of in-water light refractions that improve the visibility of pigment patterns, and uniform body postures facilitating comparison across individuals. Observers in general successfully identified matching images and ruled out non-matching ones, but some mistakes were recorded when images featured freely moving frogs. The photography methods described in this study should be easily adapted to most frog and toad species for reliable individual identification. Our study highlights that taking into account features of the natural environment of the studied species can improve individual identification by photographs.

## 1. Introduction

Capture-recapture methods enable the assessment of survival rate, growth rate, life span, population size and further parameters relevant for evolutionary-ecology studies and conservation management [1,2]. Individual identification (hereafter ‘ID’) is therefore key in population monitoring, and it can be performed either using artificial tagging methods (such as injecting transponders or colourful elastomers under the skin; [3]), genotyping [4], or exploiting natural markings [5–7]. In the latter case, individual markings are captured by photography. Pairwise comparison of the photos by a completely manual approach can become rather labour intensive as the sample size grows, but computer-assisted methods can facilitate ID in large populations across multiple years [5–7]. The latter approach saves time for the researchers by decreasing the number of photos that they have to browse in order to find a matching image, as the software provides a list of the most likely matches. While artificial tags can be lost [3,8], macroscopic colour patterns such as spots and stripes (hereafter ‘pigment patterns’) remain largely stable at least during adult life [9,10]. These stable patterns provide suitable resources for non-invasive or minimally invasive ID in various taxa including mammals, reptiles and amphibians [5–8,11]. Systematic comparisons of test databases have been conducted to assess the performance of different software that aim to reduce the time requirement of photo comparison [5,7,12–14]. While much attention has been paid to software performance, capture-recapture studies could benefit from optimized photography methods. Because species-specific natural markings evolved to be visible or provide camouflage in specific habitats combined with natural behaviour [15,16], body posture and the environment may both influence pattern recognition by conspecifics, predators, preys, and potentially by human observers as well.

**Fig 1:**
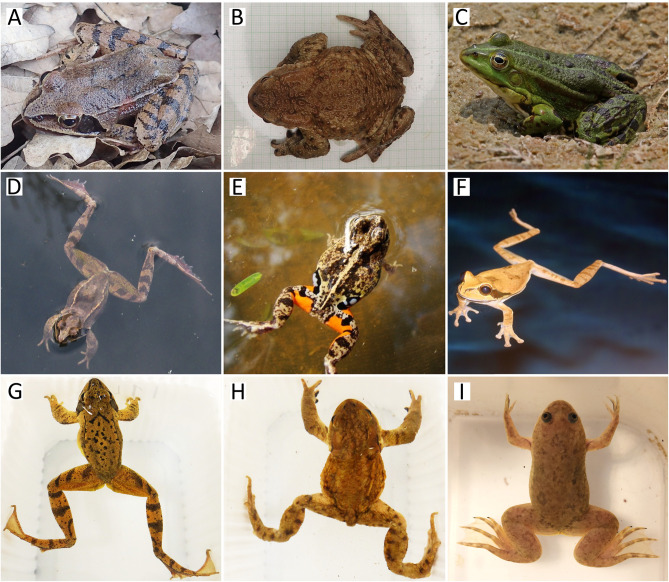
Examples of body postures of frogs and toads on land (A-C) and in water (in natural environment: D-F, or in an unfamiliar white box: G-I). While legs are typically tightly folded on land, body postures in water often provide better visibility of them. Species: agile frog (*Rana dalmatina*; **A, D, G)**, common toad (*Bufo bufo*; **B, H)**, marsh frog (*Pelophylax ridibundus*; **C)**, Colombian four-eyed frog (*Pleurodema brachyops*; **E)**, masked tree frog (*Smilisca phaeota*; **F)**, and African clawed frog (*Xenopus laevis*; **I)**. Photo credit: Nikolett Ujhegyi **(A, B, H)**, Charles J. Sharp **(C, F)**, Edina Nemesházi **(D, G)**, Luis Alberto Rueda **(E)** and Zsanett Mikó **(I)**.

Intuitively, the conditions under which photographs of animals are captured may impact pattern recognition for ID. The effect of different surrounding media (air vs. water) on pattern visibility may be especially relevant for amphibians, because individuals of most species use terrestrial as well as aquatic habitats, changing with life stages and reproductive vs. non-reproductive periods. Visual signals can be important elements of both intra- and intersexual communication during the breeding season [17]. We speculate that in species where adults are typically solitary throughout the year but aggregate in aquatic habitats for breeding [18], pigment patterns playing a role in recognizing territory neighbours, choosing mates and other types of visual communication might have evolved to be most visible in water. Therefore, it would be logical to photograph such amphibians in water in order to facilitate pattern-based ID, yet to our knowledge this approach has only been published in water-dwelling salamanders so far [8,19]. When performing photo-based ID of amphibians, it is important to take close-up images with consistent view of the region of interest. Therefore, previous studies used photographs of anurans out of water either restrained by hand [20–22], placed on their back to enable the assessment of ventral patterns [23,24], or sitting in a natural posture on a dry surface [13,14,25,26]. While this latter approach might be less stressful to the animals and can provide good view of the back and head, it is not suitable for assessing patterns across the legs, because those are usually tightly folded on both sides of the body (Fig 1). In contrast, anurans tend to take a body posture with more stretched hind legs when they are submerged in water (Fig 1); and exploiting this natural behaviour might improve ID based on dorsal patterns without having to restrain the animals. Furthermore, differential light refractions in shallow, still and clean water compared to air might improve the visibility of colour patterns by decreasing polarized light reflectance from the uneven amphibian skin surface [27]. Image quality issues regarding light conditions and reflections on frog or toad skin have been mentioned in various studies either as nuisances to avoid or as a suspected reason for reduced matching success in photo-based ID [7,12,14,20,22,25]. Yet, to our knowledge no study attempted to optimise and systematically compare the suitability of photography methods in this taxon so far, missing out on the opportunity of improving ID success in future studies.

Most pattern-recognition software has strict requirements for image layout, therefore may only be used on a well-defined area of the body and often require complex image pre-processing [7,13]. Therefore, amphibian photo-based ID studies so far have usually focused on a single body part that was deemed to feature suitably variable patterns between individuals of the focal species. Usage of dorsal patterns is more common [12–14,25,26], but ventral patterns [7,23,24], lateral patterns on one hind leg [21] and lateral lines [20] have also been used with varying success (S1 Table). However, in anurans the legs often feature at least as, if not more prominent pigment patterns than the back (e.g., majority of species in Ranidae, the large family of ‘true frogs’; but see further examples on Fig 1). Developing a photo-based identification protocol that enables the exploitation of pigment patterns across the frogs’ back as well as the dorsal surface of the legs could therefore be most beneficial; and increasing the amount of collected data may facilitate differentiation between similar-looking individuals.

In the present study, we tested different photography methods for pigment-pattern comparison across the dorsal surface of the body and legs of agile frogs (*Rana dalmatina*). These dark patterns are largely stable across ontogeny in this species and enable reliable individual identification [11]. Our aim was to assess the suitability of different image types for computer-assisted ID while enabling observers to compare as much of the body-wise patterns as possible. We chose the freely accessible HotSpotter software [28], because it proved to be suitable for whole-body comparison in a mammal [5], and in an anuran species as well (using images with limited hind-limb visibility due to sitting pose; [13]). Over the course of two study years, we created a total of 12 databases (six for males and six for females), each containing a maximum of two images per individual. The databases consisted of images featuring frogs either positioning themselves freely (without restraint) in water, or restrained by hand while photographed either with dried skin or being submerged in water. We assessed if the animal’s pose (i.e., restrained or free), surrounding medium (air or water), sex, and database size affected ID success across hundreds of individuals. For each combination of pose, medium and sex, we evaluated the ID performance of 1) the software and 2) the human observers while operating the software. We demonstrated that the combination of hand-restrained pose and water as surrounding medium improves identification success compared to the other methods.

## 2. Methods

### 2.1. Animal collection and individual identification

The agile frog is a pond-breeding brown frog widespread in Europe [29], that has been studied for its diverse developmental and behavioural responses to anthropogenic environmental changes [30,31]. We captured free-ranging agile frogs during the breeding season in 2023 and 2024 at a breeding pond near Budapest (47°33’04.3“N, 18°55’36.0”E), along a c.a. 1.5 m tall drift fence surrounding the pond, that was dug 20-cm deep underground with pitfall traps both inside and outside. The fence was standing between 03 March and 05 April in 2023, and between 06 February and 18 March in 2024. We checked the traps every morning for frogs arriving to breed or leaving the pond (hereafter, we will refer to collecting a frog from a trap as a ‘capture event’). We captured only a subset of arriving frogs in 2023 because a number of males arrived before fence building, and strong wind temporarily destroyed the fence on 11 March. We re-captured only a subset of the arriving individuals upon leaving in both years, because some frogs managed to escape the fence using climbing surfaces available in the inside, and some possibly stayed in the pond longer. We carried the animals to our nearby (ca. 700 m) experimental station in Julianna-major at the Plant Protection Institute, Budapest for photographing, and subsequently placed each individual to that side of the fence where it was heading at capture. We determined individual sex by the presence of nuptial pads (males), large belly filled with eggs (females arriving to the pond), or deflated belly and lack of nuptial pad (females leaving the pond). All frogs arriving to the breeding pond in our study were mature and confidently sexed. We assigned a unique identification number to each capture event that was photographed together with each frog.

We determined the individuals’ identities across the two years of the study based on the macroscopic colour patterns on their legs and back. We did not use artificial marking, because we recently found in an experiment that the natural colour patterns in this species remain stable for more than 15 months post-metamorphosis [11]. That study also demonstrated that photo-based ID is far more reliable than ID by visible implant elastomers or clipped toe tips in this species, reaching 98–100% ID success depending on the image-comparison process. Therefore, in the present study, we used the following approach. For each capture event, we used the images of all potential matches to identify the individual based on pigment patterns, as described in details in S1 File Section I. Briefly, in the first year, we carefully inspected the photographs of all potential matches of every frog, ensuring correct identification of the frogs in the focal population. As the dataset grew too large for this manual approach for the second year, we ensured high identification accuracy by using computer-assisted ID in two databases in parallel (containing different images from the same animals), comparing the results for each captured animal and manually double-checking all photos for all uncertain matches. We often included multiple images per individual to maximise identification success [7]. The final ID decisions were all made by a single experienced researcher. After establishing the identities of each captured individual this way, we created various subsets of matching and mismatching images (hereafter databases) as described in section 2.3, for the purpose of comparing the success of computer-assisted ID between different photography methods.

### 2.2. Photography

**Fig 2:**
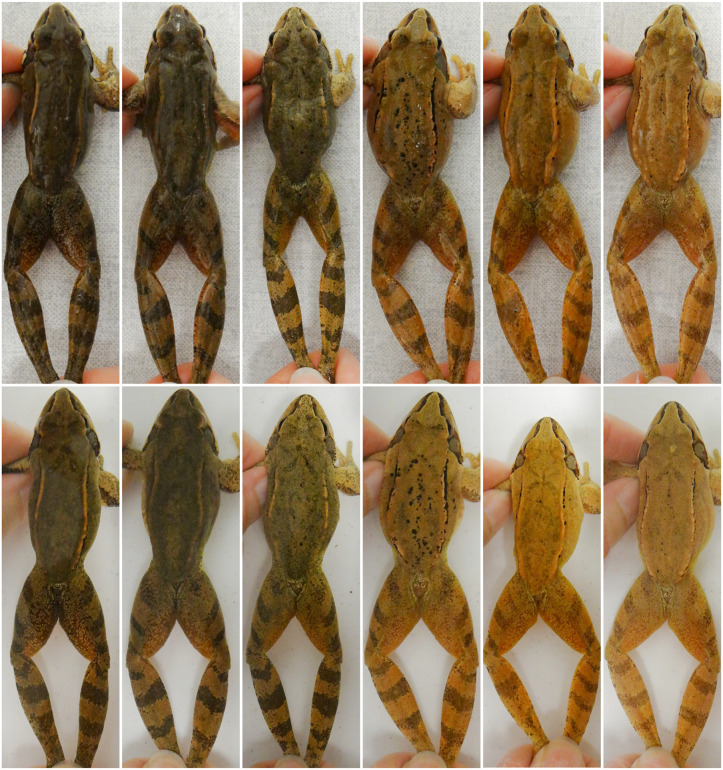
Agile frog individuals with various skin tones photographed after paper-towel drying (top) and under water (bottom). For each individual, the two images were captured a few seconds apart, using the same light source, while avoiding direct-light exposure to minimise reflections. Note how submerging in water decreases skin glossiness and shadows caused by uneven body surface, overall improving the perception of contrast between skin tone and pigment patterns. For illustration purposes on this figure, we applied the same exposure curve across all images in RawTherapee (referred to as ‘baseline’ on S2 Fig), and cropped them subsequently.

**Fig 3:**
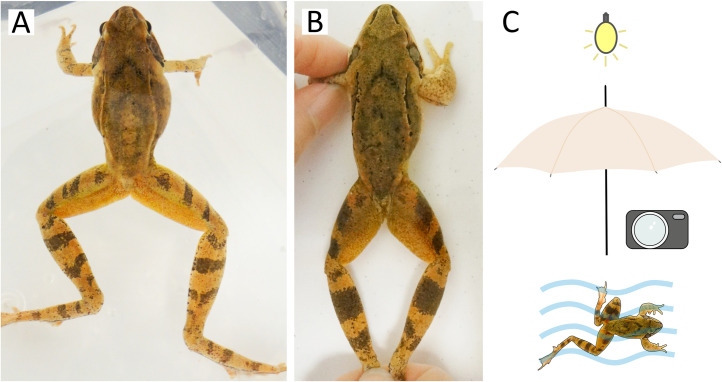
Fine-tuned photography of frogs submerged in water in 2024. The two poses are shown in water medium: ‘free’ **(A)** and ‘restrained’ **(B)**. The images were cropped to indicate the approximate coverage of the frog body that was used for ID by HotSpotter. Panel C shows visual representation of key methodological components for both image types: frogs were photographed in water, while direct light exposure was physically prevented.

We photographed agile frogs indoors, in teams of two people, where one person captured photographs (hereafter ‘photographer’) and the other handled the frogs (hereafter ‘animal handler’). Across the study, we used a Sony Cyber-shot DSC-HX200V camera (saving jpg files in 350 dpi resolution), except for 5 ‘dry-restrained’ images of arriving females in 2023, that were captured with an Olympus Tough TG-4 camera. Before photographing, each frog was washed clean by submerging into reverse-osmosis filtered and UV-sterilized water, which we reconstituted to soft water (RSW; [30]) that is ideal for amphibians. We captured three image types (i.e., using three photography methods) in total. The ‘dry-restrained’ image type (upper panel on Fig 2) featured hand-restrained frogs photographed after their skin was gently dried with a cloth or paper towel, and their hind legs being gently pulled back by the ankles (aiming for a relatively uniform body posture). When capturing the ‘water-free’ image type (Fig 3A), we placed the frogs into RSW (henceforth referred to as water) where they chose their body posture without being restrained (but posture changes were gently initiated by hand if needed, for example if the legs were tightly folded or the animal stood vertically). These two image types were captured in both 2023 and 2024. Additionally, based on our results and experiences obtained in 2023, in 2024 we decided to capture a third image type as well (i.e., ‘water-restrained’, Fig 3B), where frogs were hand-restrained in water (see also the bottom panel on Fig 2 for illustration). Learning from technical issues noted in 2023 (S1 Fig), we instructed photographers in 2024 to aim to keep the camera’s lens parallel to the back of each frog and avoid direct light exposure (i.e., lean above the animal or use an umbrella fixed on the table to obscure the ceiling light, and never use the camera’s flash; Fig 3C). We also instructed animal handlers to avoid obscuring the lower-leg patterns by their fingers, avoid twisting or stretching the hind legs to a straight posture, clean each animal in a water bath before placing it into the water where frogs are photographed, and change the latter water as needed to minimise floating debris and bubbles. Building on our experiences, we provide a detailed description of the best photography practices in S1 File Section II.

### 2.3. Image processing

We selected capture events when at least two of the three image types were taken and photography methods in general met our pre-defined methodological guidelines (see details on image selection in S1 File Section III). In those cases when more than one image was available per image type from a capture event, we chose the ones deemed most suitable for identification. With this choice, we aimed to minimize the effects of accidental photography errors on the comparison between photography methods. To this end, whenever this was possible, we preferred the image where the animal’s back and hind legs were in focus, reflections did not obscure any potential pigment patterns, and the largest possible surface of both the back and hind legs was visible on the image. When we had a single image per image type, then we used that image even if it did not meet the above criteria.

Similarly to other anurans, the hue and darkness of the skin is diverse and can change substantially in agile frogs, even within the period of a few weeks between arriving to and leaving the pond. Therefore, to increase the contrast between skin tone and pigment patterns, we applied exposure curves in RawTherapee 5.9 [32]. While these were set image by image in 2023, we used one of five pre-saved exposure curves in 2024 that we optimized for different skin tones under the here-applied light conditions (S2 Fig). In a few cases we manually altered these curves to further improve pigment-pattern visibility based on personal judgement in 2024 as well. We imported the images into HotSpotter [28] databases specified in the next paragraph, where we first set ‘chips’ (i.e., the region of interest on each image in HotSpotter) featuring the entire body of the frog (but toes may be excluded to gain a better zoom). Then, we set the orientation of the animals so the nose pointed to the left, and the vertebral column was horizontal. Because our aim was to gain as much information from each image as possible, we did not exclude the handlers’ fingertips from the ‘chips’ at the cost of cropping out visible parts of the frog body.

First we created 10 HotSpotter [28] databases (Table 1) separated by image type (‘water-free’, ‘water-restrained’ or ‘dry-restrained’), year (note that ‘water-restrained’ images were only captured in 2024) and sex. We created separate databases for the sexes because females undergo substantial body-shape changes during egg laying and might also feature behavioural differences, potentially affecting pigment-pattern visibility. When designing the databases, our aim was to enable comparison of the performance of HotSpotter as well as observers between image types, because both are key to successful computer-assisted ID. For this purpose and because database size (i.e., number of images) may affect software performance [7], within each year for each sex, the above-mentioned HotSpotter databases were identical in size for the different image types, and featured exactly one image per capture event. The detailed list and respective sizes of databases are shown in Table 1. All databases created in 2023 were of similar size. In 2024, we created larger databases from the in-water photos, and smaller ‘dry-restrained’ databases due to limited image availability for the latter. Additionally, for comparability with the ‘dry-restrained’ database in 2024, we created one subset database (named ‘water-restrained subset’) featuring ‘water-restrained’ images of the same capture events that were represented in the ‘dry-restrained’ dataset as well for each sex. Overall, this resulted in a total of 12 HotSpotter databases in our analyses, which contained images of a total of 447 male and 200 female individuals captured across the two years.

**Table 1:** False rejection rates by HotSpotter in different databases.

Database	Description	Year	N image	N individual	N match (N query)^1^	FRR_1_%	FRR_10_%	FRR_20_%
water-free 2024 female	free in water	2024	199	153	46 (66)	15.217^2^	2.174	2.174
water-free 2024 male	free in water	2024	426	374	52 (72)	23.077^2^	7.692^2^	7.692
water-restrained female	held in water	2024	199	153	46 (66)	0	0	0
water-restrained male	held in water	2024	426	374	52 (72)	0	0	0
water-restrained subset female	held in water	2024	186	145	41	0	0	0
water-restrained subset male	held in water	2024	101	52	49	0	0	0
dry-restrained 2024 female	held dry	2024	186	145	41	4.878	2.439	0
dry-restrained 2024 male	held dry	2024	101	52	49	22.449/ 4.082^3^	4.082	4.082/ 0^3^
water-free 2023 female	free in water	2023	80	69	11 (17)	18.182	0	0
water-free 2023 male	free in water	2023	108	98	8 (18)	37.5	0	0
dry-restrained 2023 female	held dry	2023	80	69	11 (17)	45.455	9.091	9.091
dry-restrained 2023 male	held dry	2023	108	98	8 (18)	50^2^	12.5^2^	12.5

^1^‘N match’ is the number of matching image pairs in the database (queries were run for each of them to calculate FRRs), and ‘N query’ is the total number of images for which queries were run by the observers (used for calculating FAR_obs_; see results in the main text).

^2^FRR_1_ differed between computers (i.e., test runs by the database creator and two observers) for the ‘water-free 2024’ database of females (ranging from 13 to 15%), while FRR_1_ and FRR_10_ differed between computers in the ‘water-free 2024’ database of males (FRR_1_ ranged between 21 and 23%, and FRR_10_ ranged between 8 and 10%) and the ‘dry-restrained 2023’ database of males (FRR_1_ ranged between 37.5 and 62.5% and FRR_10_ ranged between 0 and 12.5%). For each of these values, median across three computers is shown.

^3^For the ’dry-restrained 2024’ databases, ‘corrected’ ranks were calculated from ranks by HotSpotter (i.e., false matches with any background ‘hotspots’ excluded). FRR values differed between original and ‘corrected’ ranks only in the male database and different values are shown in the table as FRRs calculated from the original ranks/ ‘corrected’ FRR. The first number is an overestimation, while the second is an underestimation of FRRs expected for dry frogs photographed on homogenous background.

For testing purposes, each individual was represented by a maximum of two images in each of the 12 databases (i.e., one capture and one recapture image), because the number of images per individual can affect ID success [7]. For those individuals that were represented by two images in a database, we assigned one ‘chip’ (corresponding to one image) to be queried in HotSpotter, and the other ‘chip’ as its ‘true match’ that should be found. This assignment was made by the database creator, who saved the information in Excel (Microsoft Office 365; hereafter ‘query-chip table’). When running a query, HotSpotter calculated pairwise similarity scores between the ‘chips’ of the query image and all other images in the database, and ranked them accordingly with rank 1 meaning the highest similarity. To test software performance, each database was run on the database creator’s computer (the same computer across all databases), and in the ‘query-chip table’ we recorded ranks and scores assigned to ‘true matches’ and the highest-ranked non-matching images. Also, we archived visual results of HotSpotter by saving screen shots of true matching pairs of images as presented by HotSpotter, with and without circles that denoted features identified by HotSpotter as informative similarities between two images (hereafter referred to as ‘hotspots’; [33]).

After assessing the first phase of the computer-assisted ID (i.e., software performance above), we proceeded to the second phase, where human observers made ID decisions by looking at a subset of potential matches as listed by HotSpotter. To this end, databases representing each of the three image types were handed over to two out of four database operators (hereafter referred to as observers) who had not previously worked with these databases, did not participate in the identification of these individuals (section 2.1.), and used HotSpotter in 2023 for the first time. Observers were instructed to run a query in HotSpotter for each of a list of ‘chips’ (a subset of those listed in the ‘query-chip table’) and systematically screen the 10 best-ranked image pairs (in 2023; from rank = 1 to rank = 10) or the 20 best-ranked image pairs (in 2024; from rank = 1 to rank = 20), until they found a matching image. Once the observers found a match that they were confident about, regardless of the rank of the matching image, it was their decision if they kept screening or moved on to the next query. These ‘chips’ to be queried featured individuals that belonged to one of two categories: 1) were re-captured, having exactly one matching ‘chip’ in the database that should be found, or 2) had no matching ‘chip’ in the database (i.e., any assigned match would be a false positive). The observers were blind to these categories as well as to the ranks of the true matches during the tests. They recorded their ID decisions for each query in separate Excel files, and the database creator compared these results to the information in the ‘query-chip table’. Observer performance was checked in a total of eight databases across the study (each used by two of the four observers), covering all three image types: the ‘dry-restrained’ and ‘water-free’ image types in 2023, and the ‘water-restrained’ and ‘water-free’ image types in 2024. To ensure that overall observer performance with each image type was not influenced by recent experience, within each year, two observers worked first with one image type and subsequently with another, while the other two observers followed the opposite order. The observers did not test the ‘dry-restrained 2024’ and ‘water-restrained subset’ databases in 2024, because they proved to be able to reliably work with the dry image type in 2023 already, and ran queries for the ‘water-restrained’ image type in the larger database versions in 2024 (Table 1).

Finally, to better understand the differences between the reliability of different photo conditions for individual identification, we manually assessed ‘hotspot’ distribution on each matching image pair of 20 randomly chosen individuals from 2024 for each image type. The methods of this procedure are described in detail in S1 File Section V.

### 2.4. Statistical analyses

For each database, we calculated false rejection rate (FRR), which is the number of falsely rejected matches divided by the number of matching pairs present in the database for which queries were performed, expressed as a percentage [34,35]. We applied three thresholds: we accepted a match by HotSpotter if it was the image that received the highest rank (FRR_1_), or among the 10 highest ranked images (FRR_10_) or the 20 highest ranked images (FRR_20_), respectively.

‘Dry-restrained’ images in 2024 were photographed on a workbench that had light grey ornamentation on a white surface (see the upper panel on Fig 2). This may lead to overestimation of FRR if false matches received higher similarity scores due to background similarities, which may result in better ranking. To account for this potential effect, we manually checked for each query if false ‘hotspots’ were indicated on the background on those non-matching images that were ranked higher than the matching image. We are unaware of any option for removing the false ‘hotspots’ from image pairs in HotSpotter. Therefore, we calculated ‘corrected’ ranks for the true matches by excluding all those higher-ranking false matches where even a single false ‘hotspot’ was indicated on the background, and we used these ‘corrected ranks’ for calculating ‘corrected’ FRR_1_, FRR_10_ and FRR_20_ (i.e., underestimated values of FRR).

To test whether FRR varied systematically by the photography medium, body pose, sex, year, and database size, we used the data from all databases in a single generalized linear mixed-effects model with binomial error and logit link. To handle repeated measures due to the inclusion of the same individuals into different databases, we treated individual identity as a random factor. The four fixed factors and the fixed covariate (database size standardized to 0 mean and 0.5 standard deviations) were entered without interactions. Note that testing interactions between medium, pose, and year is not possible because leg patterns of frogs cannot be photographed out of water without restraint, and we do not have ‘water-restrained’ images from the first year because we came up with this method only for the second year based on our findings from the first year. To avoid the problem of separation in binomial models, we used a Bayesian implementation of linear mixed-effects models with package ‘blme’ in R 4.3.2 [36]. For defining priors, we followed Gelman’s method as recommended in [37]. We ran this model for FRR_1_, FRR_10_ and FRR_20_, and as a sensitivity analysis also for the corresponding FRRs calculated with ‘corrected ranks’. For each model, residual diagnostics were checked using the ‘DHARMa’ package. Finally, we performed sensitivity analyses by re-running all statistical models restricting the comparisons to 2024. We did not adjust the P-values because sensitivity analyses do not represent multiple testing. Detailed description of the analyses including the R code, as well as the input table are available at [33].

To assess observer performance, we calculated FRR_obs_, using the number of matching pairs present among the 10 (in 2023) or 20 (in 2024) highest ranked images on the observer’s computer as the denominator in the FRR formula, because these were the images actually searched by the observers. Additionally, we calculated the false acceptance rate (FAR_obs_) for the query results by each observer, which is the number of false matches accepted by an observer divided by the total number of screened image pairs (i.e., number of potential matches screened for each query multiplied by the number of queries assessed), expressed as a percentage.

### 2.5. Ethical statement

This study conforms to Directive 2010/63/EU and was approved by the Ethics Committee of the Plant Protection Institute. All procedures were permitted by the Environment Protection and Nature Conservation Department of the Pest County Bureau of the Hungarian Government (PE/EA/295–7/2018, PE/EA/00270–6/2023, PE-06/KTF/07949–6/2023, PE-06/KTF/00754–8/2022, PE-06/KTF/00754–9/2022). When handling the frogs, we paid attention not to cause harm and unnecessary stress to the animals by keeping handling time as short as possible. We avoided introducing infectious diseases to the studied breeding population by disinfecting all equipment with 96% ethanol before the start of animal handling. The present study was carried out within one breeding population of high density (ca. 600 individuals breeding in a ca. 180 m^2^ pond) and the animals often gathered in the pitfall traps in high numbers (multiple dozens per trap spending hours in close physical contact). Due to the unavoidable physical contact between large numbers of frogs during pitfall-trapping, we deemed it unjustified from an infection-prevention perspective to change the water between each individual, and to make wearing single-use gloves mandatory for the participants who handled the frogs. However, paper towels and water baths were replaced after every few animals, and all containers were washed clean and dried at the end of each day. Furthermore, the participants were obliged to disinfect their hands before starting animal handling each day and not to use any hand lotion or cream, to protect the sensitive frog skin.

## 3. Results

Regardless of sex, all true matches received rank = 1 by HotSpotter in the ‘water-restrained’ databases and the ‘water-restrained subset’ (FRR_1_ = 0%), while only fewer true matches received rank = 1 in all other databases (FRRs ranging from 4 to 50%; Table 1). Overall, FRR_10_ and FRR_20_ were relatively low for all databases (Table 1). Statistical analyses showed that, with all three rank thresholds, the usage of both hand-restrained pose and water medium significantly improved FRR compared to the frogs taking a free pose and/or being photographed out of water (Table 2); sex and database size had no significant effect on FRRs (Table 2). For FRR_1_, the methodological changes applied in 2024 resulted in significant improvement compared to 2023, with similar tendencies for FRR_10_ and FRR_20_ (Table 2). All these results were supported by the sensitivity analyses, where ranks were corrected for potential false ‘hotspots’ in the 2024 ‘dry-restrained’ images, with two exceptions: 1) the effect of year was significant for FRR_20_ as well, and 2) both FRR_1_ and FRR_20_ increased with database size, with a similar tendency for FRR_10_ (Table 2). Results of the sensitivity analyses restricted to 2024 confirmed that both the water medium and the hand-held pose significantly reduced FRR in 5 out of 6 models (the effect of medium was not significant when the corrected ranks of the first 20 matches were used). Similarity scores of the matching images and the highest-ranked non-matching images for each query are available in the result table on Figshare [33].

**Table 2:** Results of the Bayesian generalized linear mixed-effects models with FRR values reflecting software performance, calculated either based on the original ranks for all databases (FRR), or based on corrected ranks in the ‘dry-restrained 2024’ but original ranks in all the other databases (Corrected FRR).

	FRR			Corrected FRR		
Model coefficients	Rank 1	Rank 10	Rank 20	Rank 1	Rank 10	Rank 20
**Intercept**	6.03 ± 2.28**	−0.76 ± 2.68	−1.72 ± 2.66	10.77 ± 3.94**	−0.76 ± 2.68	2.48 ± 4.12
**Medium (water vs. dry)**	−8.5 ± 1.67***	−7.3 ± 2.34**	−6.63 ± 2.25**	−11.54 ± 2.59***	−7.3 ± 2.34**	−9.18 ± 3.08**
**Pose (restrained vs. free)**	−7.33 ± 1.51***	−5.41 ± 1.85**	−5.31 ± 1.83**	−10.07 ± 2.44***	−5.41 ± 1.85**	−7.54 ± 2.54**
**Sex (male vs. female)**	1.09 ± 1.1	0.28 ± 1.67	0.54 ± 1.79	−3.02 ± 1.79^†^	0.28 ± 1.67	−1.23 ± 2.9
**Year (2024 vs. 2023)**	−4.85 ± 1.52**	−2.51 ± 1.48^†^	−2.54 ± 1.51^†^	−7.9 ± 2.18***	−2.51 ± 1.48^†^	−5.9 ± 2.73*
**Database size**	0.86 ± 0.7	2.6 ± 1.43^†^	2.02 ± 1.4	6.03 ± 1.37***	2.6 ± 1.43^†^	6.78 ± 2.76*

Each value is a model-estimated coefficient with ± standard error, on logit scale. The intercept refers to the FRR for the images of females in 2023 with dry medium and free posture, at average database size. All other coefficients are non-standardized effect sizes for the difference of each category relative to the intercept. Significant effects are denoted by asterisks (*P < 0.05, **P < 0.01, ***P < 0.001) and marginally non-significant effects (0.05 < P < 0.095) are denoted by dagger symbols (†).

In the observer performance tests, out of the 16 database-observer combinations, the observer FRR was non-zero in only one case, and FAR was non-zero in two cases. In the ‘water-free 2024’ databases, three out of the four observers successfully identified all matching image pairs when those were provided among the 20 highest-ranked matches by HotSpotter (FRR_obs2–4_ = 0%), while one observer missed one such match in the ‘water-free 2024’ database of females (FRR_obs1_ = 2.222%). None of the observers made such mistakes with the ‘water-restrained’ databases, nor with the ‘water-free 2023’ and ‘dry-restrained 2023’ databases (FRR_obs1–4_ = 0%). Regardless of database type, observers never recorded non-matching images as matching in 2024 (FAR_obs1–4_ = 0%); despite that two out of the four observers made such mistakes with the ‘water-free 2023’ database type a year earlier (FAR_obs1_ = 1.176% and FAR_obs2_ = 0.588%, respectively).

**Fig 4:**
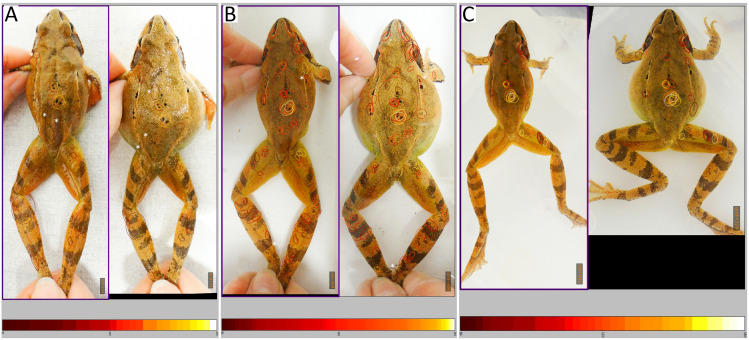
Distribution of ‘hotspots’ displayed by HotSpotter. Image pairs feature the same female after (on the let) and before (on the right) egg deposition in the ‘dry-restrained’ **(A)**, ‘water-restrained’ **(B)** and ‘water-free’ **(C)** databases of females in 2024. The colour of the ovals denoting ‘hotspots’ indicate similarity strength scores increasing from dark red to bright yellow as shown on the colour bars under the images (A: 50-150, B: 80-240, C: 60-180; explanation taken from [6]). Although all ‘hotspots’ are located on the animal in each panel, those ovals in one image that have no matching oval at the same location in the other image denote false ‘hotspots’ (we marked these by asterisks).

Qualitative assessment of the distribution of ‘hotspots’ on matching image pairs revealed that HotSpotter recognized pigment patterns across the entire frog body (S3 Fig). However, specificities of the photo conditions, such as leg posture and light refractions altered the outcome (see Fig 4 for examples). The number of ‘hotspots’ on pigment patterns on the legs was lowest in the ‘water-free’ databases, due to varying leg postures across the images. Misleading ‘hotspots’ caused by shadows were common in the ‘dry-restrained’ databases but never occurred when the animals were photographed in water. As expected, false ‘hotspots’ on the background occurred only in the ‘dry-restrained’ databases, where the background was a faintly ornamented bench surface. We found ‘hotspots’ that were likely caused by colour shades rather than clear pigment patterns in all database types, but they were relatively more common when the animals were restrained by hand (S3 and S4 Figs).

## 4. Discussion

Overall, matching images received significantly better ranks in HotSpotter when frogs were photographed in water instead of out in the dry. We assume that the main reason for this difference was that, when frogs were photographed dry, shadows generated by back muscles and uneven skin surfaces obscured some pigment patterns and induced false ‘hotspots’ (see S3 Fig). Due to differential light refractions, these shadows vanished when frogs were submerged in water, facilitating pigment-pattern comparison for HotSpotter. In this respect, our findings correspond with a previous report where HotSpotter performed somewhat better with in-water photos than with out-of-water photos of sea turtles [6]. These results show that the choice of medium can matter for the success of photographic ID in agile frogs and sea turtles, raising the possibility that ID success for other aquatic or semi-aquatic species may also be improved by taking in-water images. More generally, environmental light conditions such as water turbidity for aquatic animals [38,39], or forest versus open-field light for terrestrial animals [16], may be worth taking into account when trying to find the best setup for ID-photographing. Our results did not support the idea that allowing the animals to take a natural body posture in water would facilitate ID success. Instead, the more-or-less uniform pose of hand-restrained frogs permitted better performance for both HotSpotter and the human observers, once we had eliminated the technical problems associated with dry photography we detected in the first year. Our results highlight that photographing the frogs under water in a standardized hand-restrained position can enable very fast ID whereby the observers have only to check the first-ranked match in HotSpotter (FRR_1_ = 0) to find out the ID of the queried image. Moreover, the observers in our study made no mistakes with this image type (FRR_obs1–4_ = 0 and FAR _obs1–4_ = 0). Nevertheless, after technical improvements from the first to the second year, we found reasonably good ID success with the ‘dry-restrained’ and the ’water-free’ methods as well, although with these image types we found that the 10 best-ranked matches of HotSpotter should be checked for each query to keep FRR below 10%. By checking the 20 best-ranked images, FRR can be kept close to zero with the optimized ‘dry-restrained’ method, meaning that the amount of time needed for finding the right match with nearly 100% certainty is up to 20 times longer compared to the ‘water-restrained’ method.

Survival rate and abundance are two key metrics in population monitoring, and to assess how reliable a database is for calculating these, it is essential to calculate FRR and FAR as well. False rejection leads to underestimation of survival rate and overestimation of abundance, while false acceptance has the opposite effects. FRR values by HotSpotter calculated in our ‘water-free 2024’ and ’dry-restrained 2024’ databases were comparable to those reported in the most successful of previous anuran ID studies (S1 Table). In contrast, when restrained individuals were photographed in water, all true matches received the highest rank in HotSpotter (FRR_1_ = 0). To our knowledge, no previous study with similarly large anuran databases demonstrated such high success for photo-based ID. Previously Matthé et al. [7] found that success rate increases (i.e., FRR decreases) in smaller subset databases when using various other software, and we found qualitatively similar correlation between database size and FRR using HotSpotter. The largest databases used in our study consisted of 426 images, corresponding with the relatively larger database sizes for which FRR or interchangeable metrics were reported in previous anuran studies (S1 Table). Furthermore, FRR decreases as the number of images per individual increases in a database [7]. Because we included a maximum of one matching image pair for each individual in our databases, including multiple images would be expected to further improve performance of our methods, especially for the ‘water-free’ databases where different images of the same frog feature different leg postures. In fact, anuran databases with the lowest FRRs published so far contained more than two images of certain individuals [7,14,25,26]; this contrast further highlights that the zero FRR found here when restrained frogs were photographed in water is outstanding. Nevertheless, it is important to keep in mind that ID success may decrease over longer time scale (e.g., due to aging and injuries) when carrying out photo-based ID, potentially causing increased FRR for the older individuals.

The image types we tested here by HotSpotter are expected to be suitable for further platforms as well [5,40]. Observers in general were able to identify matching images captured by each photography method, although FRR_obs_ and FAR_obs_ results taken together from 2023 and 2024 show that at least some observers make mistakes with the ‘water-free’ image type. Previous studies rarely assessed FAR in anuran photo-based identification, but in those few cases where such attempts were made, the reported false acceptance was either 0% [14,23], or it was lower than 1% [20]. However, FAR might be much higher in some cases [41]. In our study, FAR was low, confirming high suitability of the tested methods for individual identification based on unique dorsal pigment patterns across the body and the legs (0% for ‘restrained’ and 0–1% for ‘water-free’ photos).

In this study we used photographs featuring agile frogs. Because various other anuran species show similarities in pigment-pattern distribution across the body, and similar behaviour in water (Fig 1), we expect that the conclusions of our study would stand for further anurans as well. We propose that photographing anurans in water would be a beneficial practice for various monitoring programs, not only because improved pigment-pattern visibility can lead to more reliable ID, but also because these procedures may be less stressful for the frogs than photographing them out of water. Amphibians are most comfortable when their skin is wet, whereas they can remain underwater for much longer than needed for taking a photograph, because they can exchange gas through their skin [42]. We noticed no specific reaction by agile frogs to being submerged for the ‘water-restrained’ photography (e.g., males continued without interruption the pulsing vocalization that they often use when grabbed by other males or humans). It is likely that letting them move freely in water is even less stressful for them. Therefore, usage of the ‘water-free’ image type may be a preferred choice when working with sensitive species or small juveniles (i.e., to minimise handling time, and to prevent potential injuries when handling small and fragile animals), even at the expense of a somewhat slower or slightly less accurate ID compared to the ‘water-restrained’ method.

In practice, infrastructural limitations and differences in manpower requirement may constrain what photography method can be used in a specific project. For example, while a single person can photograph freely-moving animals alone, photos featuring the entire body of hand-restrained frogs require the assistance of a second person as well. A further challenge is when images captured by varying cameras and under varying conditions are used in the same study, making it harder for the software (and potentially also for the human observers) to compare them [12,14,20,24]. Here we used a single camera and standard artificial light conditions indoors, which may not be feasible for all studies, but still there is a lot researchers can do to improve image quality. Based on our experiences from the present study, photo-based ID can be improved by avoiding glare caused by direct light exposure, keeping the water clean and choosing appropriate container size and water level (our detailed practical advices are described in S1 File Section II). Keeping the above viewpoints in mind, applying these photography methods to other species should be fairly straightforward.

In conclusion, comparison of the ranks received by true matches across different HotSpotter databases demonstrated that 1) photographing frogs in water instead of with towel-dried skin improved ID, and 2) images of hand-restrained frogs in a standardized position submerged in water outperformed the other image types, but 3) photographing agile frogs moving freely in water also demonstrated to be a reasonable option for ID. We propose that the methods tested here, which enable the assessment of patterns across the entire body including legs, can facilitate a refined and more reliable photo-based ID by improving the quality of data compared to conventionally used photography methods. Finally, our results highlight that taking into account the natural conditions under which colour patterns evolved may facilitate photo-based individual identification for animal monitoring.

## Supporting information

S1 File Additional information on the methods and results. (DOCX)

S1 Table False rejection rates (FRR) in previous computer-assisted individual identification studies with anuran amphibians. Published results by three thresholds are shown: matching was considered successful if at least one matching image 1) received the highest rank (FRR_1_), 2) was among the 10 highest-ranked images (FRR_10_) or 3) was among the 20 highest-ranked images (FRR_20_).
(DOCX)

S1 Fig Examples of a male (A, B) and a female (C, D) individual photographed in hand on dry (A, C) and freely in water (B, D) in 2023. Direct exposure to artificial ceiling light caused strong glare on some individuals despite being dried by paper towel (A), and some frogs took postures that made photographing more difficult, such as standing vertically in the water (D) instead of floating or laying horizontally (B). Note the small bubbles and pieces of dirt in the water over the frog body (B, D).
(PDF)

S2 Fig Examples of different skin tone and pigment-pattern strength combinations and the results of applying different exposure curves in RawTherapee in 2024. The setting deemed best in terms of pigment-pattern visibility for each photo is marked with a green circle. Short name for each curve is shown on top (the leftmost version is the original photo); the exposure curve marked with asterisk was created for the special case when a pale animal is featured on an unusually bright photo. Also, note the lack of bubbles and pieces of dirt in the water over the frog body.
(PDF)

S3 Fig Visual assessment of ‘hotspot’ distribution on matching image pairs in different databases. The order of ten randomly-chosen male and female individuals (one row per individual) is the same across database types ‘dry-restrained’ (top), ‘water-restrained’ (center) and ‘water-free’ (bottom), respectively. Ovals displayed by HotSpotter denoting ‘hotspots’ were categorized by a single human observer.
(JPG)

S4 Fig Illustration of strong (A) and mild (B) changes of distinct-shade spots on the back. Within each panel, the upper and lower image features the same animal in 2023 and 2024, respectively.
(JPG)
