# Supplementary material for: Placing anurans in water can improve photo-based individual identification
Source: PLoS One. 2026 Jan 30;21(1):e0341460. doi: 10.1371/journal.pone.0341460 (PMC12857928; doi:10.1371/journal.pone.0341460)
Supplement: S1 Table — Published results by three thresholds are shown: matching was considered successful if at least one matching image 1) received the highest rank (FRR1), 2) was among the 10 highest-ranked images (FRR10) or 3) was among the 20 highest-ranked images (FRR20). (DOCX) [file pone.0341460.s002.docx]

**S1 Table.** **False rejection rates (FRR) in previous computer-assisted individual identification studies with anuran amphibians.** Published results by three thresholds are shown: matching was considered successful if at least one matching image 1) received the highest rank (FRR_1_), 2) was among the 10 highest-ranked images (FRR_10_) or 3) was among the 20 highest-ranked images (FRR_20_)

| **Study** | **Species** | **N images** | **N image/ individual** | **Software** | **FRR_1_ (%)** | **FRR_10_ (%)** | **FRR_20_ (%)** |
| --- | --- | --- | --- | --- | --- | --- | --- |
| Matthé et al (2017) ^1^ | *Bombina variegata* | varying: 500-4000 | varying (2-10) | multiple | 2.3-19.6 | 1.2-11.4 | - |
|  |  | 4063 | varying (2-10) | multiple | 3.1-19.6 | 1.7-11.4 | - |
| Patel & Das (2020) | *Amolops formosus* | 301 | varying (2-4) | HotSpotter | 5.6 | - | - |
| Caorsi et al (2012) ^1^ | *Melanophryniscus cambaraensis* | 492 | - | Wild-ID | 26 | - | - |
| Davis et al (2020) ^1^ | *Rhinella diptycha* | 109 | varying (2-3 or more) | Wild-ID | - | - | 0? ^3^ |
| Dawson et al (2021) ^1^ | *Pelophylax lessonae* | 465 | - | multiple | 45.9-59.2 | - | - |
| Burgstaller et al (2021) ^2^ | *Bufotes viridis* | 200 | 2 | multiple | c.a. 40-60 | - | - |
|  |  | 200 | 2 | HotSpotter | c.a. <10 | - | - |
| Morrison et al (2016) | *Anaxyrus baxteri* | 130 | - | HotSpotter and Wild-ID | - | - | 56.6-76.2 |
| Aevarsson et al (2022) | *Xenopus longipes* | 48 | 2 | Wild-ID | - | - | 0-17 |
|  |  | 20 | 2 |  | - | - | 100 |
| Kim et al (2017) | *Dryophytes japonicus* | - | - | Wild-ID | - | - | 25 |
|  |  | 213 ? | - |  | - | - | 100 |

^1^ FRR was derived from the reported ‘success rate’ (100-‘success rate’).

^2^ FRR was derived from ‘correctly identified images %’ shown on Figure 3 (100-‘correctly identified images %’).

^3^ Cut-off criteria for ‘success’ are not clear, so the ‘100% success rate’ might reflect other than true matches present between ranks 1 and 20.

**References**

Aevarsson, U., Graves, A., Carter, K. C., Doherty-Bone, T. M., Kane, D., Servini, F., Tapley, B., & Michaels, C. J. (2022). Individual identification of the lake Oku clawed frog (*Xenopus longipes*) using a photographic identification technique. *Herpetological Conservation and Biology*, *17*(1), 67–75.

Burgstaller, S., Gollmann, G., & Landler, L. (2021). The green toad example: A comparison of pattern recognition software. *North-Western Journal of Zoology*, *17*(1), 96–99.

Caorsi, V. Z., Santos, R. R., & Grant, T. (2012). Clip or Snap? An evaluation of toe-clipping and photo-identification methods for identifying individual southern red-bellied toads, *Melanophryniscus cambaraensis*. *South American Journal of Herpetology*, *7*(2), 79–84. https://doi.org/10.2994/057.007.0210

Davis, H.-P., VanCompernolle, M., & Dickens, J. (2020). Effectiveness and reliability of photographic identification methods for identifying individuals of a cryptically patterned toad. *Herpetological Conservation and Biology*, *15*(1), 204–211.

Dawson, J., Panter, C. T., & Zeisset, I. (2021). Comparisons of image-matching software when identifying pool frog (Pelophylax lessonae) individuals from a reintroduced population. *Herpetological Journal*, *31*(1), 55–59. https://doi.org/10.33256/31.1.5559

Kim, M. Y., Borzée, A., Kim, J. Y., & Jang, Y. (2017). Treefrog lateral line as a mean of individual identification through visual and software assisted methodologies. *Journal of Ecology and Environment*, *41*(1), 42. https://doi.org/10.1186/s41610-017-0060-1

Matthé, M., Sannolo, M., Winiarski, K., Spitzen - van der Sluijs, A., Goedbloed, D., Steinfartz, S., & Stachow, U. (2017). Comparison of photo-matching algorithms commonly used for photographic capture–recapture studies. *Ecology and Evolution*, *7*(15), 5861–5872. https://doi.org/10.1002/ece3.3140

Morrison, T. A., Keinath, D., Estes-Zumpf, W., Crall, J. P., & Stewart, C. V. (2016). Individual identification of the endangered Wyoming Toad *Anaxyrus baxteri* and implications for monitoring species recovery. *Journal of Herpetology*, *50*(1), 44–49. https://doi.org/10.1670/14-155

Patel, N. G., & Das, A. (2020). Shot the spots: a reliable field method for individual identification of *Amolops formosus* (Anura, Ranidae). *Herpetozoa*, *33*, 7–15. https://doi.org/10.3897/HERPETOZOA.33.E47279
