# Supplementary figures and images for: Placing anurans in water can improve photo-based individual identification
Source: PLoS One. 2026 Jan 30;21(1):e0341460. doi: 10.1371/journal.pone.0341460 (PMC12857928; doi:10.1371/journal.pone.0341460)

A

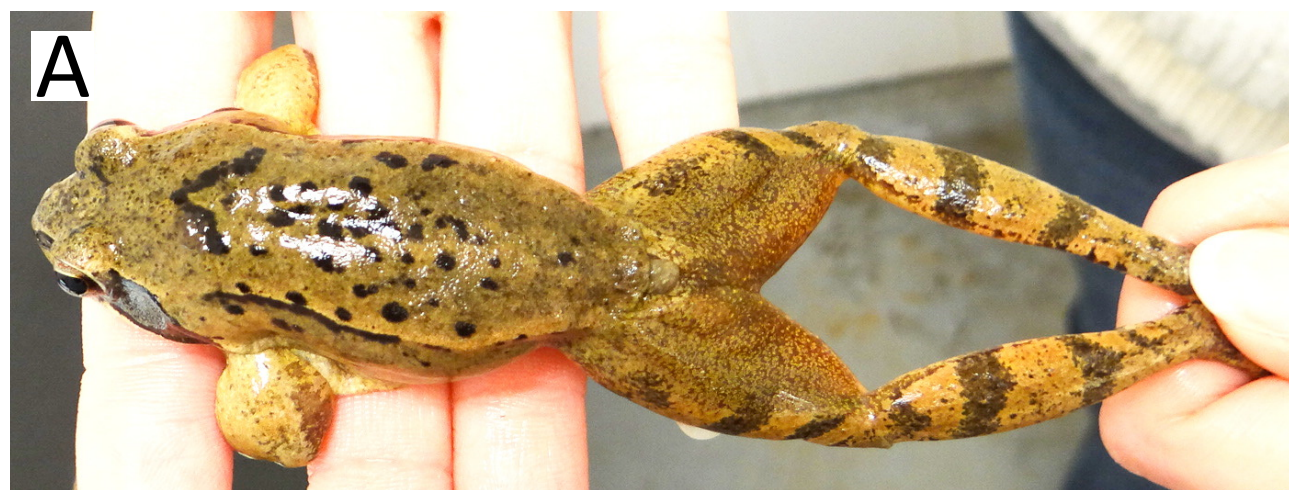

C

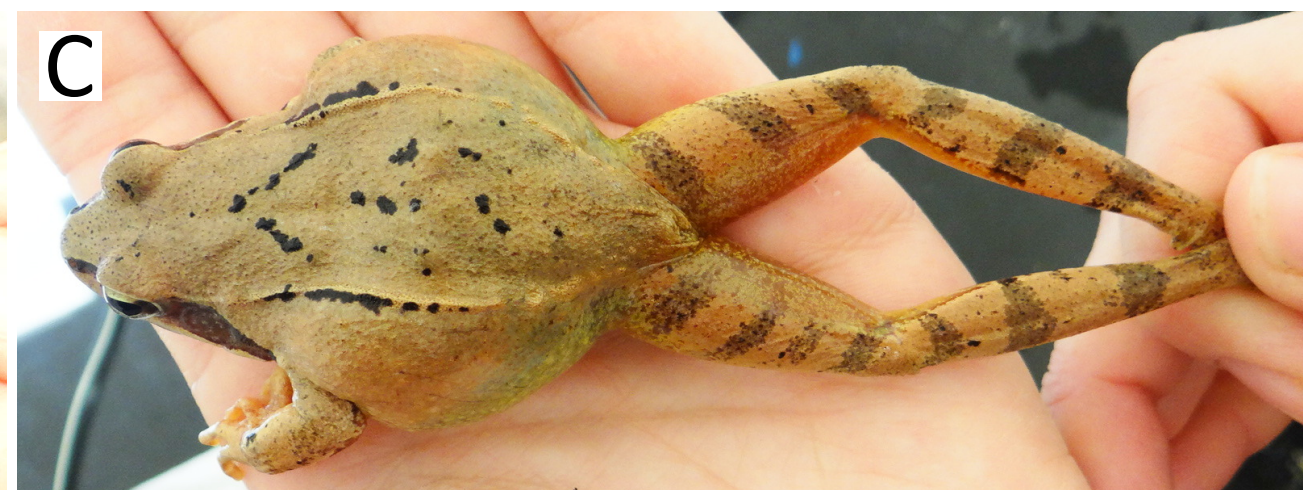

B

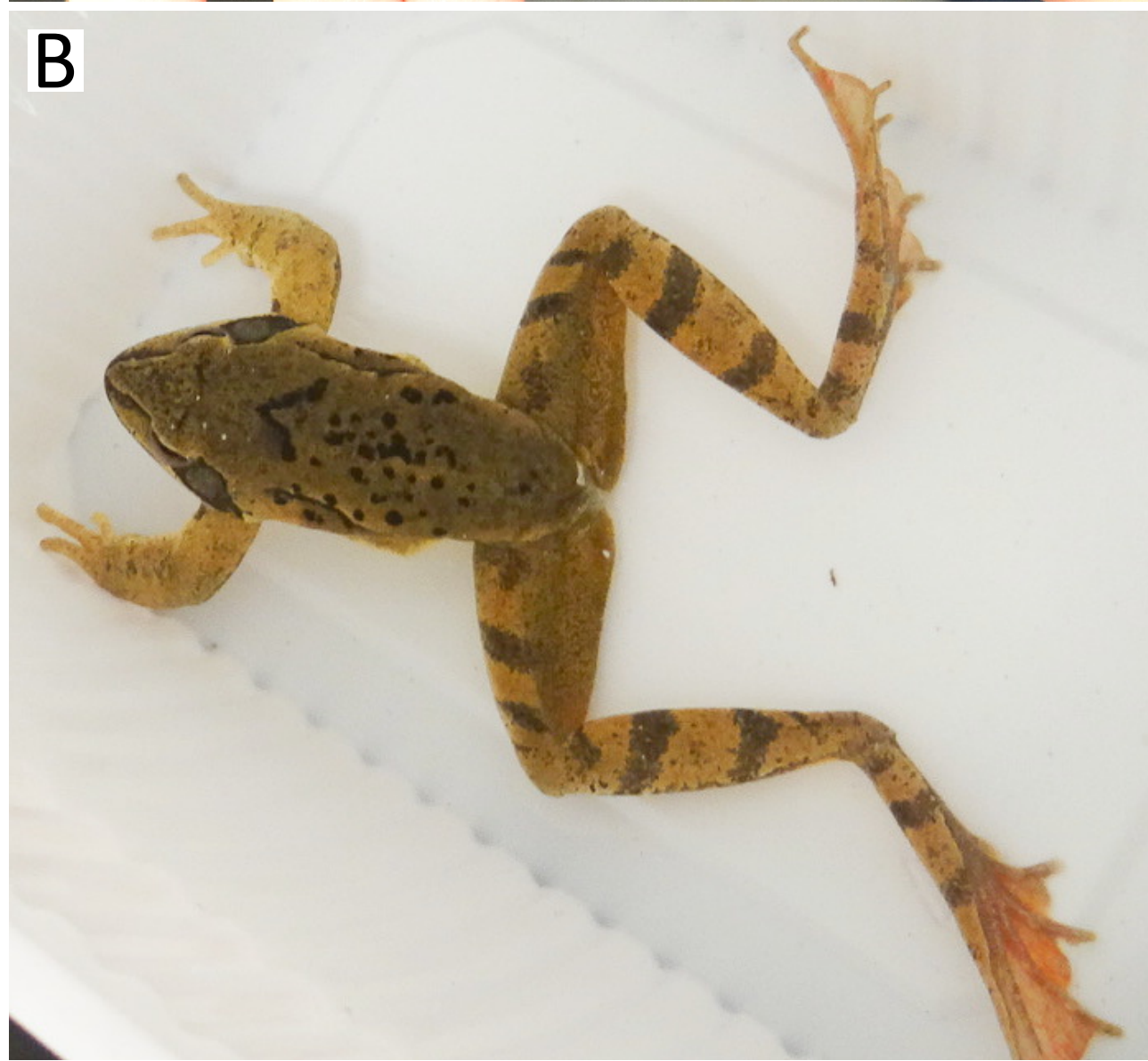

D

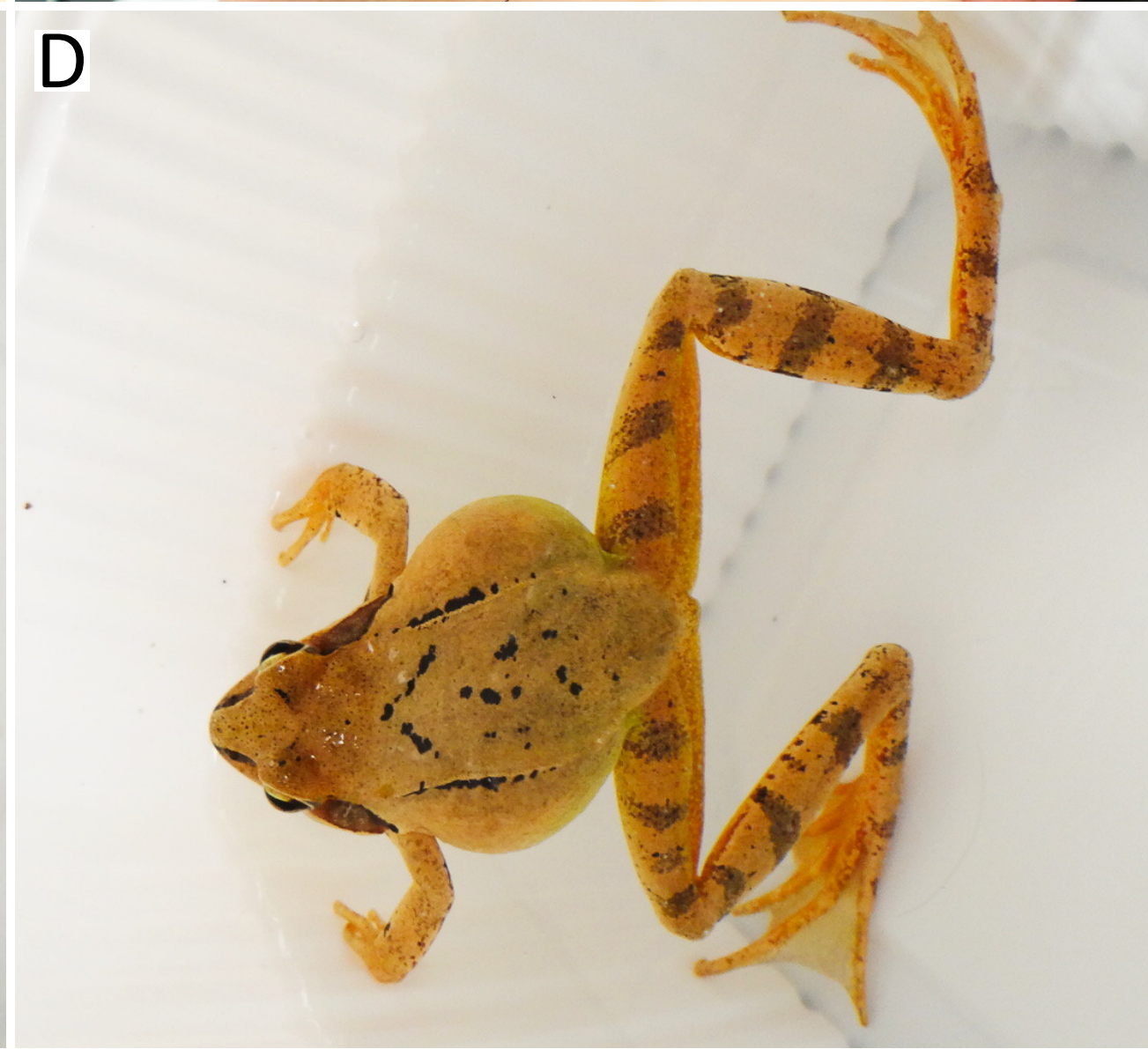

Supplement: S1 Fig — Direct exposure to artificial ceiling light caused strong glare on some individuals despite being dried by paper towel (A), and some frogs took postures that made photographing more difficult, such as standing vertically in the water (D) instead of floating or laying horizontally (B). Note the small bubbles and pieces of dirt in the water over the frog body (B, D). (PDF) [file pone.0341460.s003.pdf]

Pale\*

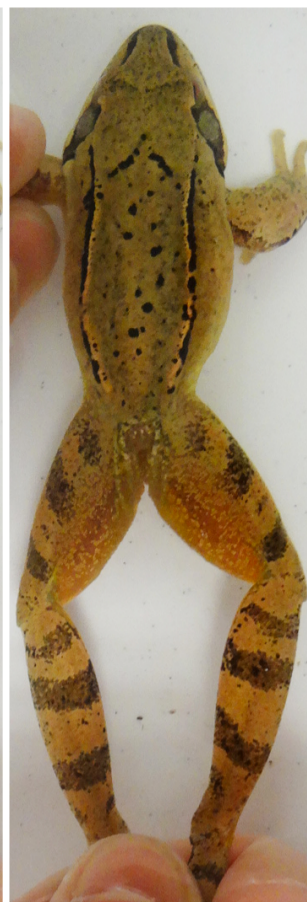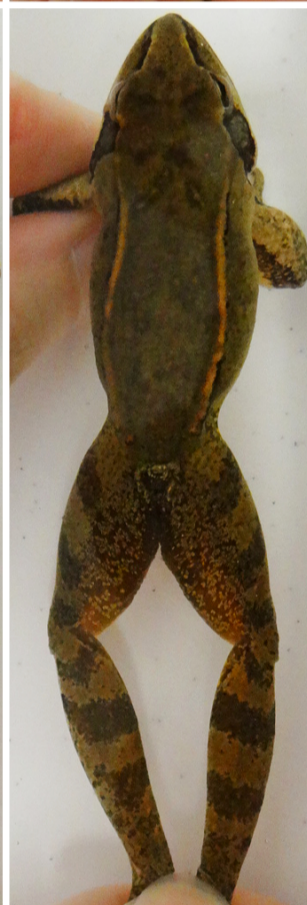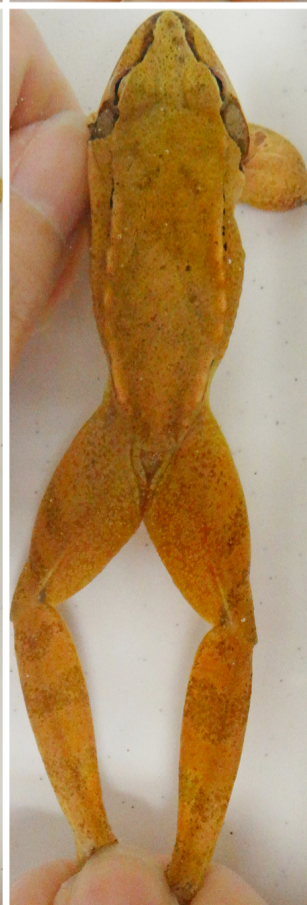

Supplement: S2 Fig — The setting deemed best in terms of pigment-pattern visibility for each photo is marked with a green circle. Short name for each curve is shown on top (the leftmost version is the original photo); the exposure curve marked with asterisk was created for the special case when a pale animal is featured on an unusually bright photo. Also, note the lack of bubbles and pieces of dirt in the water over the frog body. (PDF) [file pone.0341460.s004.pdf]

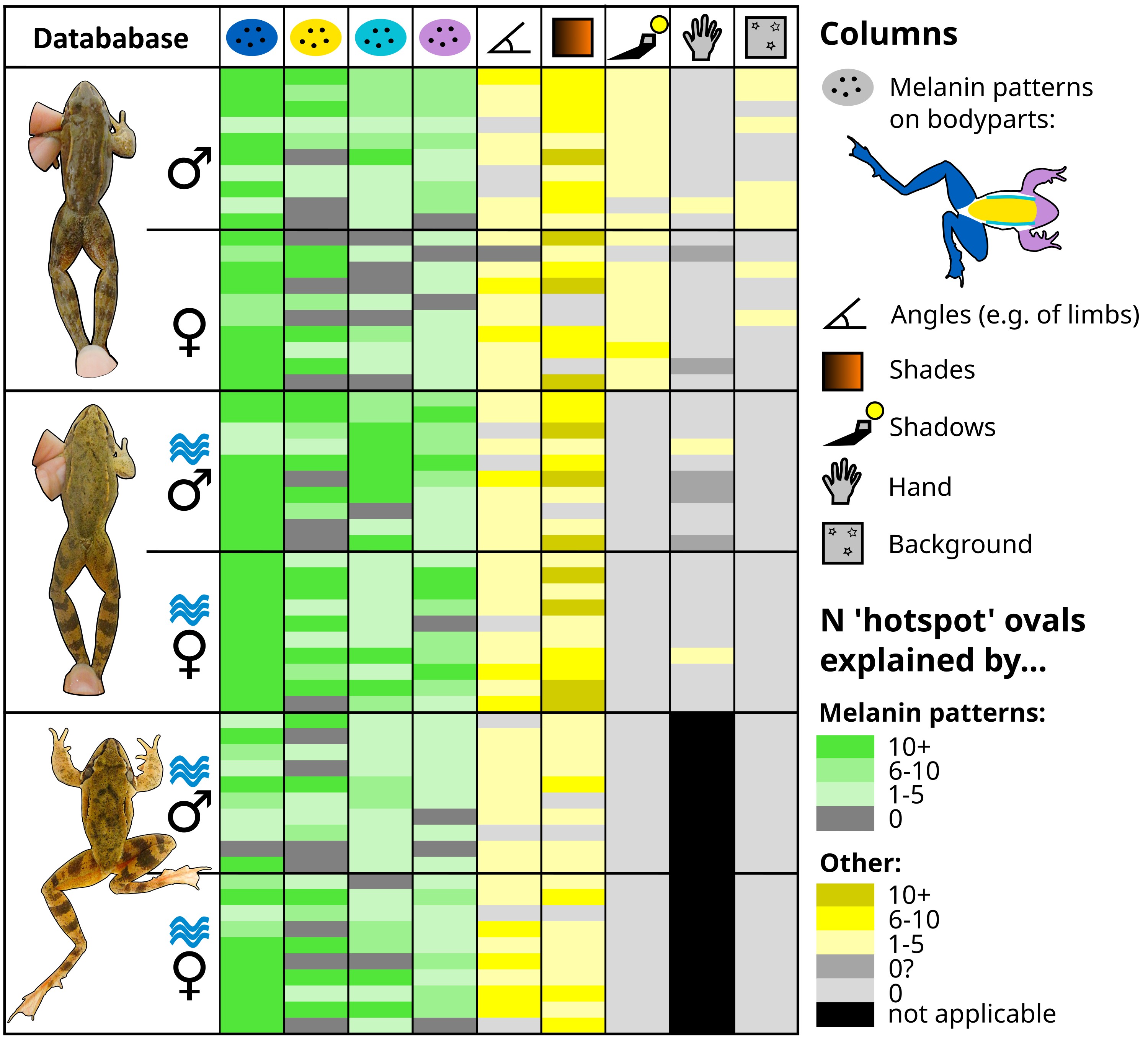

Supplement: S3 Fig — The order of ten randomly-chosen male and female individuals (one row per individual) is the same across database types ‘dry-restrained’ (top), ‘water-restrained’ (center) and ‘water-free’ (bottom), respectively. Ovals displayed by HotSpotter denoting ‘hotspots’ were categorized by a single human observer. (JPG) [file pone.0341460.s005.jpg]

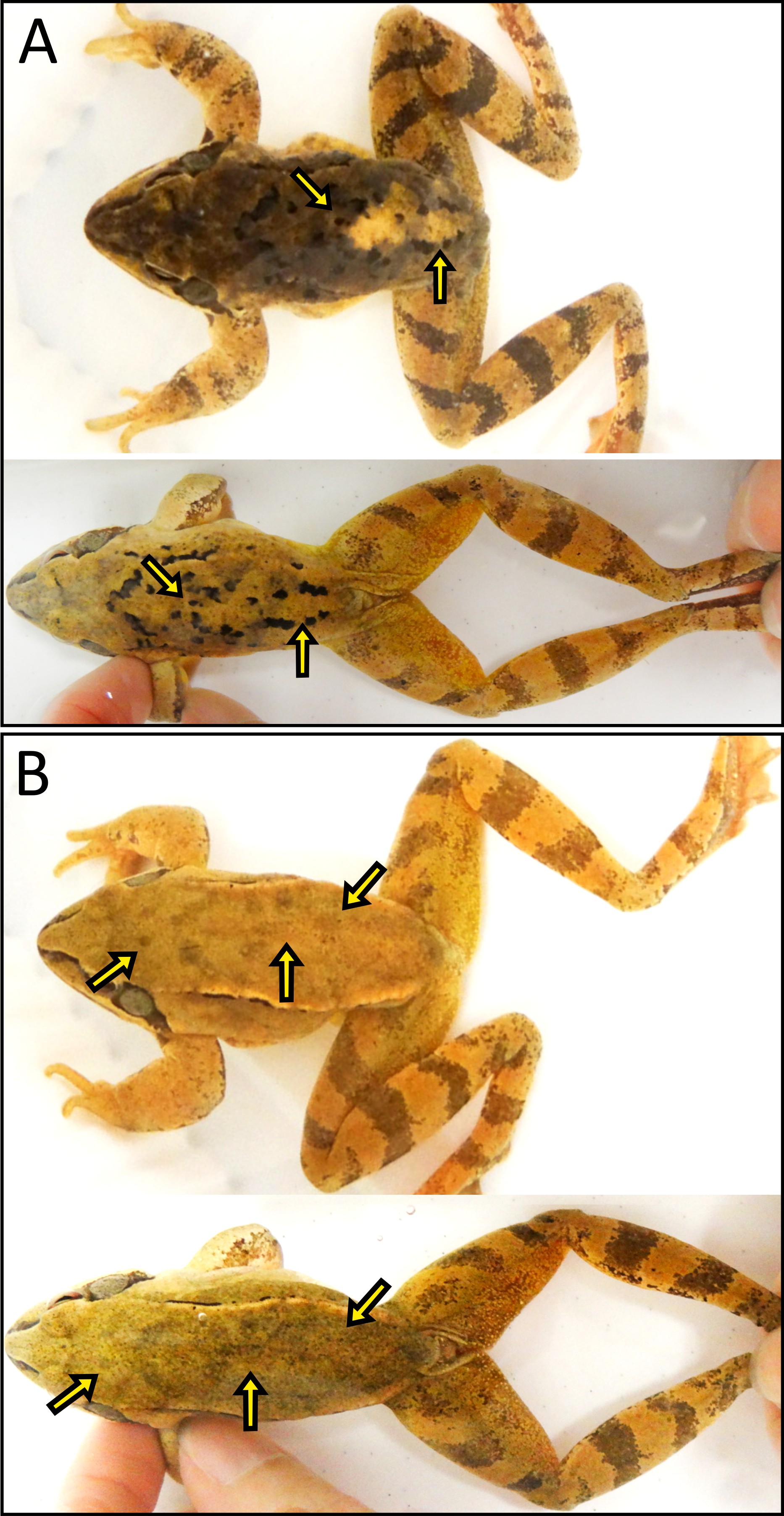

Supplement: S4 Fig — Within each panel, the upper and lower image features the same animal in 2023 and 2024, respectively. (JPG) [file pone.0341460.s006.jpg]
